# Profiling of host genetic alterations and intra-tumor microbiomes in colorectal cancer

**DOI:** 10.1016/j.csbj.2021.05.049

**Published:** 2021-06-04

**Authors:** Shujiro Okuda, Yoshifumi Shimada, Yosuke Tajima, Kizuki Yuza, Yuki Hirose, Hiroshi Ichikawa, Masayuki Nagahashi, Jun Sakata, Yiwei Ling, Nobuaki Miura, Mika Sugai, Yu Watanabe, Shiho Takeuchi, Toshifumi Wakai

**Affiliations:** a Division of Bioinformatics, Niigata University Graduate School of Medical and Dental Sciences, 1-757 Asahimachi-dori, Chuo-ku, Niigata 951-8510, Japan; b Division of Digestive and General Surgery, Niigata University Graduate School of Medical and Dental Sciences, 1-757 Asahimachi-dori, Chuo-ku, Niigata 951-8510, Japan; c Division of Cancer Genome Informatics, Niigata University Graduate School of Medical and Dental Sciences, 1-757 Asahimachi-dori, Chuo-ku, Niigata 951-8510, Japan; d Division of Medical Technology, Niigata University Graduate School of Health Sciences, 2-746 Asahimachi-dori, Chuo-ku, Niigata 951-8518, Japan

**Keywords:** Tumor microbiome, Colorectal cancer, Fusobacterium, Campylobacter, Mutational signature

## Abstract

Some bacteria are symbiotic in tumor tissues, and metabolites of several bacterial species have been found to cause DNA damage. However, to date, the association between bacteria and host genetic alterations in colorectal cancer (CRC) has not been fully investigated. We evaluated the association between the intra-tumor microbiome and host genetic alterations in 29 Japanese CRC patients. The tumor and non-tumor tissues were extracted from the patients, and 16S rRNA genes were sequenced for each sample. We identified enriched bacteria in tumor and non-tumor tissues. Some bacteria, such as *Fusobacterium*, which is already known to be enriched in CRC, were found to be enriched in tumor tissues. Interestingly, *Bacteroides*, which is also known to be enriched in CRC, was enriched in non-tumor tissues. Furthermore, it was shown that certain bacteria that often coexist within tumor tissue were enriched in the presence of a mutated gene or signal pathway with mutated genes in the host cells. *Fusobacterium* was associated with many mutated genes, as well as cell cycle-related pathways including mutated genes. In addition, the patients with a high abundance of *Campylobacter* were suggested to be associated with mutational signature 3 indicating failure of double-strand DNA break repairs. These results suggest that CRC development may be partly caused by DNA damage caused by substances released by bacterial infection. Taken together, the identification of distinct gut microbiome patterns and their host specific genetic alterations might facilitate targeted interventions, such as modulation of the microbiome in addition to anticancer agents or immunotherapy.

## Graphical abstract

**Figure:**
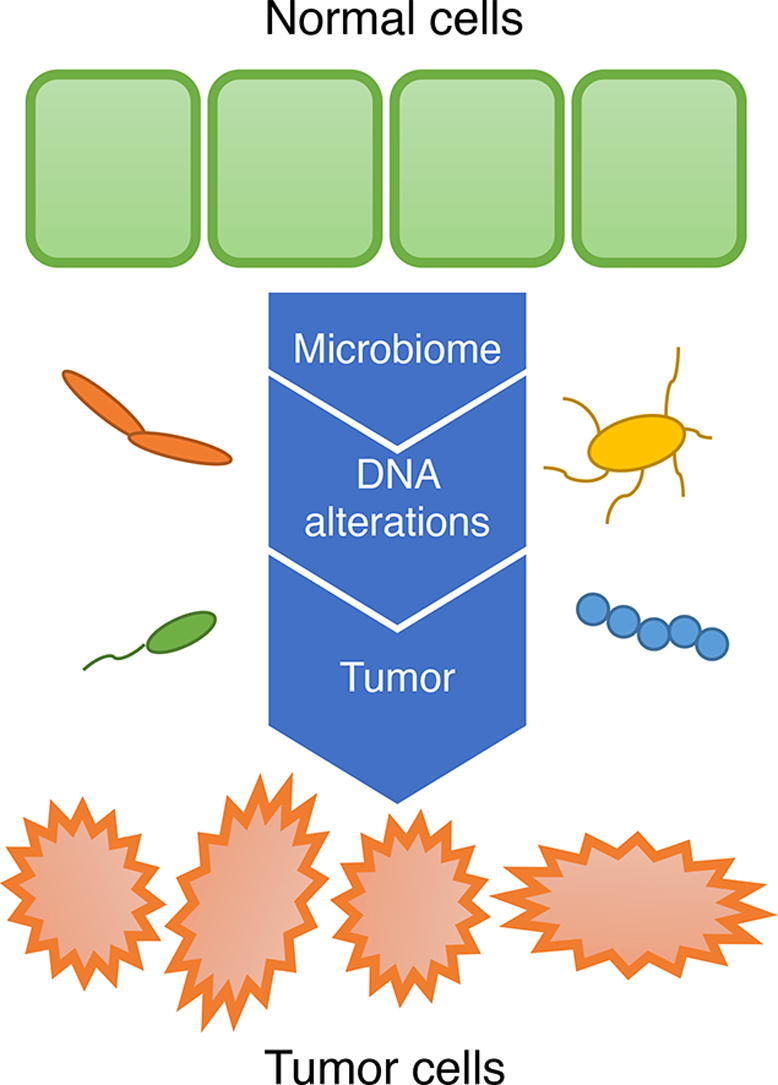

## 1 Introduction

Colorectal cancer (CRC) is one of the most common cancers worldwide [1], and it is a heterogeneous disease with varying morphological features, clinical outcomes, and responses to anticancer agents and immunotherapy [2], [3], [4]. Colorectal carcinogenesis represents a heterogeneous process associated with various sets of molecular alterations that are influenced by the gut microbiome, diet, and environment [5]. The human intestinal microbiome regulates various aspects of health, and alterations in it can contribute to disease [6]. Advances in metagenomic analyses have revealed that changes in the intestinal microbiome, which can affect metabolism and immune function [7], [8], [9], may initiate and promote CRC [10], [11].

Recently, it has been found that certain bacteria are symbiotic in tumor tissues [12], and have the potential to affect the efficacy of anticancer agents [13] and immunotherapy [14], [15] in CRC. Modification of the gut microbiome is regarded as a therapeutic strategy for cancer treatment, and efforts are currently underway to enhance therapeutic responses or abrogate treatment-associated toxicity via modulation of the gut microbiome [16]. For example, dietary interventions can be used to modulate the gut microbiome of patients receiving cancer therapy [5].

Various bacteria have been associated with the development of CRC, including Bacteroides, Fusobacterium, Salmonella, Escherichia, and Campylobacter, and the mechanisms involved in carcinogenesis have been elucidated in some bacteria. *Fusobacterium nucleatum* is known to be symbiotic in the tumor tissue, and it has been suggested that *F*. *nucleatum* increases the proliferation of cancer cells by promoting the activation of the β-catenin and Wnt pathways via E-cadherin [17]. Metabolites of several bacterial species have been found to cause DNA damage. *Campylobacter jujuni* produces cytolethal distending toxin (CDT), a genotoxin that has DNAse activity and causes DNA double-strand breaks [18]. Enterotoxigenic Bacteroides fragilis (ETBF) and genotoxic *pks* + Escherichia *coli*, which produce a cyclomodulin toxin called colibactin, were also associated with DNA damage in CRC [19], [20]. We now have strong evidence that the presence of specific microorganisms in the microbiota is associated with various stages of CRC development, and that these microorganisms can initiate tumor formation and contribute to tumor growth [21].

In context of the clinical implications of the composition of microbiome, we hypothesize that the study of gut microbiome-induced genetic alterations may guide the development of novel treatment strategies for CRC. Burns et al. demonstrated statistically significant association between loss-of-function mutations in CRC tumor genes, and sifts in the abundances of specific sets of bacterial taxa in the gut microbiome, using microbiome profiling and whole-exome sequencing in 44 pairs of tumors and matched normal tissues [22]. However, it is unclear whether changes in the composition of microbiota can cause specific and actionable genetic alterations, and the clinical implications of these changes for treatment strategies for CRC remains to be elucidated.

We hypothesized that distinct gut microbiome patterns are associated with specific genetic mutations in CRC. Identification of distinct gut microbiomes and their specific genetic alterations in tumor tissue could facilitate targeted interventions, such as modulation of the microbiome, in addition to anticancer agents or immunotherapy. Hence, we investigated genetic alterations in tumor tissue using a 415-gene panel for testing designed for tumor agnostic treatment. We aimed to evaluate the association between intra-tumor microbiota and host genetic alterations detected by gene panel testing in CRC.

## 2 Methods

### 2.1 Patients

This retrospective analysis was performed in accordance with the Declaration of Helsinki and other relevant guidelines and regulations. The Ethics Committee of the School of Medicine, Niigata University, approved the study protocol (G2015-0816), and written informed consent was obtained from all patients. We enrolled 29 Japanese patients with stage I–IV CRC according to the American Joint Committee on Cancer guidelines, 8th edition [2], [3], [4], who agreed with the study protocol and had received primary tumor resection between 2015 and 2016 at Niigata University Medical and Dental Hospital. We included 6 patients with MSI-H and 23 patients with MSS in this analysis. Patients with CRC with familial adenomatous polyposis or inflammatory bowel disease were excluded. This retrospective cohort study did not include any differences in nutrition. The patient information is shown in Supplementary Table 1.

### 2.2 Next-generation sequencing (NGS) for detecting genetic alterations in CRC

As previously described [23], [24], [25], [26], [27], formalin-fixed, paraffin-embedded (FFPE) samples were used for NGS, and genetic alterations in the tumor tissue were evaluated in 29 patients with stage I–IV CRC. We did not analyze non-tumor tissues for genetic alterations in this study. All sample preparations, NGS, and bioinformatics analyses were performed in a CLIA/CAP-accredited laboratory (KEW, Cambridge, MA, USA) [28]. Briefly, hematoxylin and eosin-stained sections were used to assess tumor content, ensuring > 50% tumor content in the tissue samples. The unstained sections were macroscopically dissected for tumor content enrichment. DNA was extracted using a BioStic FFPE Tissue DNA Isolation Kit (Mo Bio Laboratories, Inc., Carlsbad, CA, USA). First, 50–150 ng DNA fragment libraries were prepared and enriched with a panel of 415 genes (CANCERPLEX version 3.0). Library construction was performed according to the manufacturer’s instructions. Illumina MiSeq and NextSeq (San Diego, CA, USA) platforms were used to achieve an average sequencing depth of 500 × . A proprietary bioinformatics platform based on GATK Best Practices [29] and MuTect tool kit [30], and knowledge database, were used to process genomic data and identify genomic abnormalities, including single nucleotide variants (SNVs), small insertions/deletions (indels), copy number variations (CNVs), and translocations [30], [23]. To assess the somatic state of the mutations in a tumor-only environment, we adopted a filtering strategy similar to one previously published [31], with some modifications. In other words, mutations present in the dbSNP, 1000 Genomes, and ExAC databases were lowered in priority (AF > 1%). The model was then fitted using the frequency of the alleles of each mutation, to determine whether the mutation was likely to be germline cell heterozygous or somatic. A 10% allelic fraction threshold for SNVs and indels, as well as thresholds of > 2.5-fold and < 0.5-fold for gain and loss, respectively, were used.

### 2.3 16S rRNA gene sequencing

We extracted the tumor and non-tumor regions from fresh tissue samples from all 29 CRC cases. We took the non-tumor tissue, which was macroscopically diagnosed as “non-tumor,” from within 3 cm of the tumor. Generally, the lesion located adjacent to the tumor is possibly affected by ischemia and/or inflammation induced by the tumor, so we defined the lesion as “non-tumor”, not “healthy”. After the fixation of the resected specimen, we confirmed the area of “non-tumor” tissue to be microscopically non-tumor tissue. DNA was extracted from fecal samples using an automated DNA extraction machine (GENE PREP STAR PI-480, Kurabo Industries Ltd., Osaka, Japan) according to the manufacturer’s instructions. 16S rRNA gene sequencing was performed for each sample using the Mykinso® technology developed by Cykinso Inc., (Tokyo, Japan), which included DNA extraction and subsequent 16S rRNA paired-end sequencing using the Illumina MiSeq platform [32]. The FASTQ file thus obtained was processed to join the forward and reverse reads to a single read per sample using fastq-join [33] with default settings. Next, low quality sequences were excluded using QIIME version 1.9 [34], and chimera sequences were removed using USEARCH [35]. Relative abundance was calculated after detecting OTUs at 97% identity for the filtered sequence data using QIIME's pick_open_reference_otus command. The number of reads in each step is listed in Supplementary Table 2.

### 2.4 Contaminant filtering and re-calculation of relative abundance

Recent advances have shown that bacteria exist in a variety of tumor tissues, and these studies have suggested that contamination may occur during the process of tissue sampling and subsequent processing. We removed the contaminants from the species obtained in this study, based on species identified in a previous study reported by Neijman et al. [12]. The species name that matched to the contaminant species name were filtered out, after which we re-calculated the relative abundance of the remaining species to be normalized to 1.0. The relative abundance at the upstream taxonomy levels was re-estimated based on the re-calculated relative abundance at the species level.

### 2.5 Enrichment analysis

To identify the bacteria enriched in the tumor area, the relative abundance of each taxon in tumor and non-tumor tissues was tested using a one-sided Wilcoxon rank sum test. Taxa with P < 0.05 were extracted for further analysis. Supplementary Table 3 includes the list of taxa filtered out in the contaminant filtering step related to this statistical test. In addition, to explore the association of bacterial taxa with gene alterations or signal pathways with altered genes, we performed another enrichment analysis. We calculated the differences by subtracting the relative abundance of the bacterial taxa in non-tumor tissue from that in tumor tissue, in order to take into account the cases of zero values. To confirm whether a bacterium was enriched in the presence or absence of a gene alteration, patients were divided into groups based on the presence or absence of a gene alteration on the host side. The differences in their relative abundances were tested using a one-sided Wilcoxon rank-sum test. Similarly, patients were divided into groups based on the presence or absence of a gene alteration in the set of genes involved in a signal pathway on the host side, and the differences in their relative abundances were tested using a one-sided Wilcoxon rank sum test. R (https://www.r-project.org) was used for the calculations, along with the exactRankTests library.

### 2.6 Regression analysis

In order to test the association between clinical data and each bacterial genus, we conducted multivariate generalized linear regression using the differences in the abundance of each genus in tumor and non-tumor tissues as response variables and clinical data extracted from Supplementary Table 1 as covariates.

To identify significantly related altered genes or signal pathways with altered genes that coexist in bacterial species, tests of associations were conducted between mutated status and the difference in the relative abundance of bacterial taxa in tumor and non-tumor tissues of the 29 samples, which were fitted by non-parametric linear regression models based on Siegel median estimators, because the method could effectively avoid the influence of outliers [36]. This regression was performed using the difference values ​​of each taxon in patient populations with or without gene alterations. Furthermore, patients were divided according to the presence or absence of alterations in genes participating in signal transduction pathways defined in the KEGG Network database, [37] and the same statistical test was performed. FDR-adjusted P-values (Q-values) were calculated for each taxon [38]. Alteration frequencies of less than 15% and less than five patients with altered genes were filtered out in the enrichment analysis.

### 2.7 Mutational signatures

Mutational signatures of 29 CRC tumor tissues were analyzed as follows: each SNV was classified in a matrix of the 96 possible substitutions, based on the sequence context comprising the nucleotides 5 and 3 to the position of the single nucleotide mutation [39]. Mutational signatures were extracted using non-negative matrix factorization analysis with the ‘Somatic Signatures’ library, and plotted with the ‘ggplots’ library (http://ggplot2.org/) in R.

### 2.8 Hierarchical clustering analysis

To compare patterns of the relative abundances or P-values of enriched taxa and altered genes obtained from the enrichment analysis, hierarchical clustering was performed using the Euclidean distance and Ward’s method in R.

## 3 Results

### 3.1 Comparison of microbiomes in tumor and non-tumor tissues

**Fig. 1:**
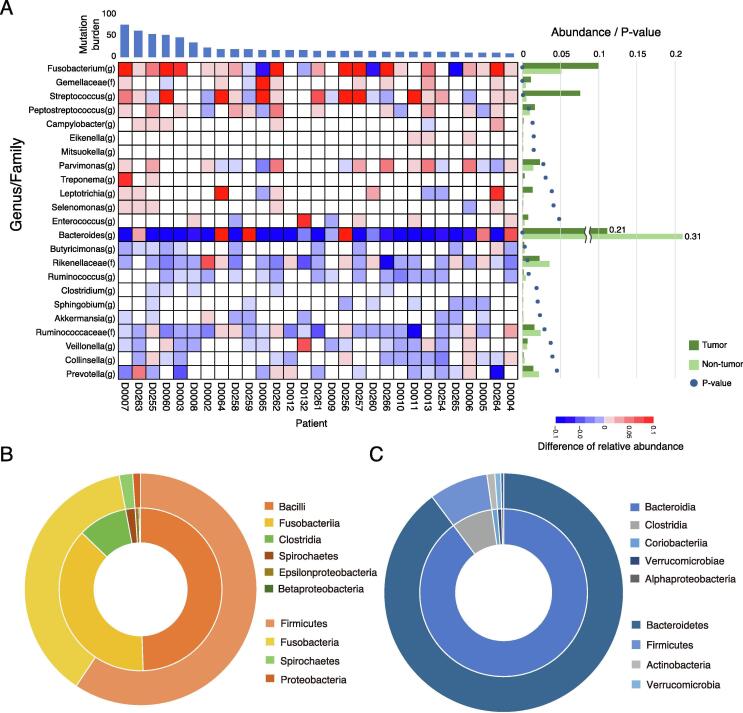
Differences between tumor and non-tumor-enriched taxa. (A) Heatmap shows difference between the relative abundance of each genus in tumor and non-tumor tissues. When the relative abundance in tumors is greater or lesser than in non-tumor samples, the cells are shown in red or blue, respectively. Genera positively enriched in tumor tissue and non-tumor tissue (P < 0.05, Wilcoxon rank sum test, one-sided) were extracted. The averages of the relative abundances are shown as a bar chart in the right of the figure. The green bars indicate the abundance in tumor tissue, and the rig green bars indicate the abundance in non-tumor tissue. The p-values are also indicated as circles in the chart. Distributions for the taxa are shown in (A) enriched in tumor tissue (Fusobacterium(g) – Enterococcus(g)), and (B) enriched in non-tumor tissue (Bacteroides(g) – Prevotella(g)). The same are shown in (C) as a pie chart. Outer circle indicates phylum level and inner circle indicates class level distributions. (For interpretation of the references to colour in this figure legend, the reader is referred to the web version of this article.)

16S rRNA gene sequencing was performed on the tumor and non-tumor tissue samples, to determine the relative abundance of each taxon for each sample at the genus level. Subsequently, a taxonomy enrichment analysis was performed to compare tumor and non-tumor tissues (Fig. 1 and Supplementary Table 3). Twelve genera, including *Fusobacterium* (P < 0.001), *Peptostreptococcus* (P < 0.01), and *Campylobacter* (P < 0.05), were found to be positively enriched in tumor tissue. In contrast, 11 genera, including *Bacteroides* (P < 0.001) and *Clostridium* (P < 0.05), were found to have a lower relative presence in tumor tissues compared to non-tumor tissues. Genera of phylum *Firmicutes*, including *Fusobacterium*, were enriched in tumor tissue (Fig. 1B), and genera of phylum *Bacteroidetes*, including *Bacteroides*, were enriched in non-tumor tissue (Fig. 1C). In addition, we performed statistical tests to study the associations between the clinical information and each genus (Supplementary Table 4). *Treponema* was found to be associated with tumor mutation burden (TMB) with high abundance in tumor tissue (P < 0.0003), but the association of others was not significant.

### 3.2 Gene alteration and tumor-enriched microbiome

**Fig. 2:**
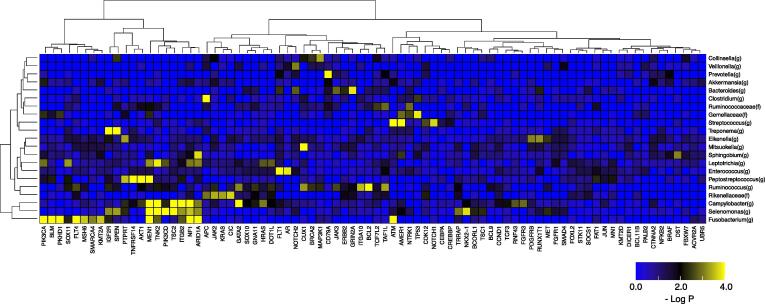
Enrichment analysis of taxonomy with host gene alterations. Heatmap shows P-values of enrichment analysis between genus-level taxonomy and gene alterations. For the taxa found in the enrichment analysis comparing tumor and non-tumor samples, further enrichment analysis for the presence or absence of gene alterations, including nonsynonymous SNVs, Indels, CNVs, and translocations, was performed in patients. Alteration frequencies of > 15% and > 5 patients with altered genes were filtered out, and the hierarchical clustering was then performed. The heatmap is colored according to significance (negative log P-value) of the enrichment, where yellow indicates a higher degree of enrichment. (For interpretation of the references to colour in this figure legend, the reader is referred to the web version of this article.)

We investigated how bacterial genera that were enriched and depleted in the tumor tissue were associated with the presence or absence of host gene alterations. After dividing patients according to the presence or absence of host gene alterations, we conducted enrichment analysis for each taxon in the patient groups. The differences between relative abundances of bacterial genera in tumor and non-tumor tissues were calculated and used for enrichment analysis. Hierarchical clustering analysis of the taxa and altered genes was performed based on the enrichment results at the genus level (Fig. 2 and Supplementary Table 5). Fusobacterium was associated with the most number of altered genes (11 genes with P < 0.001, Q < 0.01), including *ATM* (P < 0.001, Q < 0.001) and *PIK3CA* (P < 0.001, Q < 0.001). *Streptococcus* showed the most significant association with alteration in *ATM* (P < 0.0001, Q < 0.0001), *Treponema* was significantly associated with alterations in *SPEN* (P < 0.001, Q < 0.001) and *IGF2R* (P < 0.001, Q < 0.001), and *Peptostreptococcus* was significantly associated with alterations in *MEN1* (P < 0.001, Q < 0.001) and *AKT1* (P < 0.001, Q < 0.001), *Campylobacter* was associated with alterations in *TNK2* (P < 0.001, Q < 0.001) and *GATA2* (P < 0.001, Q < 0.001), and *Selenomonas* was associated with alterations in *MEN1* (P < 0.001, Q < 0.001) and *PIK3CD* (P < 0.001, Q < 0.01).

### 3.3 Signal transduction pathways and tumor-enriched microbiome

**Fig. 3:**
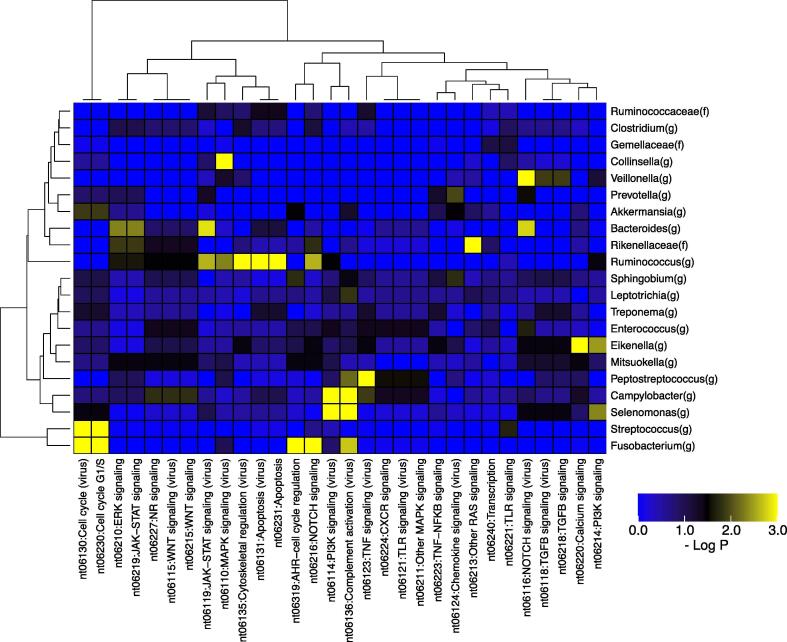
Enrichment analysis of associations of taxa with alterations in genes involved in signal transduction pathways. Heatmap shows P-values for enrichment analysis of genus-level taxonomy and signal transduction pathways. For the taxa found in the enrichment analysis of comparison of tumor and non-tumor samples, further enrichment analysis for the presence or absence of gene alterations in signal transduction pathways defined in the KEGG Network was performed. The pathways with fewer than two genes and fewer than five patients with altered genes were filtered out, and hierarchical clustering was then performed. The heatmap is colored according to significance (negative log P-value) of the enrichment, where yellow indicates a higher degree of enrichment. (For interpretation of the references to colour in this figure legend, the reader is referred to the web version of this article.)

To study the relationship between the host signal transduction system abnormalities and symbiotic bacteria that are positively enriched in the tumor tissue, enrichment analysis was performed after dividing the patients into groups based on the presence of at least one gene alteration, such as SNVs, indels, CNVs, or translocations, in a set of genes involved in a signaling pathway defined in the KEGG Network database. The differences in relative abundances of bacterial genera between tumor and non-tumor tissues in the patients divided in the above way were then used for enrichment analysis. As a result, some genera were found to be positively enriched in patients with alterations in genes involved in specific signal transduction pathways (Fig. 3 and Supplementary Table 6). *Campylobacter* and *Selenomonas* were associated with gene alterations in the PI3K signaling (virus) (nt06114) (P < 0.001, Q < 0.01) and complement activation (virus) (nt06136) (P < 0.001, Q < 0.01) signal transduction pathways. *Fusobacterium* and *Streptococcus* were associated with gene alterations in the cell cycle systems (nt06230: cell cycle G1/S and nt06130: cell cycle (virus)) (P < 0.001, Q < 0.001). *Fusobacterium* was also significantly associated with other signaling pathways, including NOTCH signaling (nt06216) (P < 0.001, Q < 0.001) and AHR − cell cycle regulation (nt06319) (P < 0.001, Q < 0.01). *Peptostreptococcus* was significantly associated with TNF signaling (virus) (nt06123) (P < 0.001, Q < 0.001) and *Ruminococcus* was associated with apoptosis (nt06231) (P < 0.001, Q < 0.001).

### 3.4 Mutational signatures of host genomes with presence or absence of *Campylobacter*

**Fig. 4:**
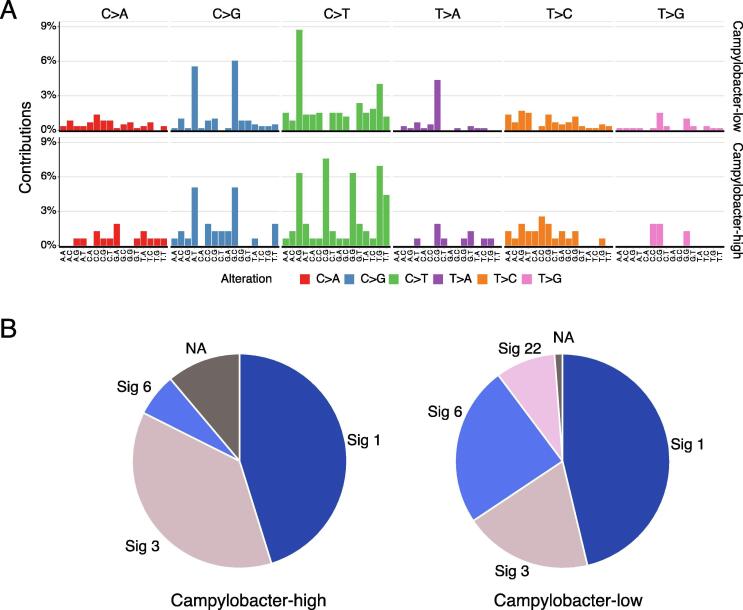
Mutational signatures with *Campylobacter* infection. (A) Mutational spectra in *Campylobacter*-high and *Campylobacter*-low patients based on the differences of relative abundances of *Campylobacter* in tumor and non-tumor tissues. Note that samples without *Campylobacter* were classified as low. (B) Mutational signature distributions of *Campylobacter*-high and *Campylobacter*-low patients. Patients with hypermutation were removed from the mutational signature analysis to avoid bias for the number of gene mutations.

Different mutational processes generate different combinations of mutation types, termed “Mutational Signatures” [40], [41]. If the host DNA damage is caused by a bacterial infection, it may leave a mutational signature. *Campylobacter* has recently been shown to produce cytolethal distending toxins that cause DNA double-strand breaks in the host DNA [18]. We performed mutational signature analysis by classifying patients into two groups, in which the relative abundance of *Campylobacter* was higher or lower in tumor tissues than in non-tumor tissues. As a result, signatures 1, 3, 6, and 22 were detected. Signature 1 is associated with the age of cancer diagnosis, Signature 3 is a mutational signature associated with the failure of DNA double-strand break repair by homologous recombination, Signature 6 is associated with defects in DNA mismatch repair, and Signature 22 is related to cancer samples exposed to aristolochic acid in the COSMIC database [40], [41]. We found that the *Campylobacter*-high group had a much higher percentage of Signature 3 than the *Campylobacter*-low group (Fig. 4). These findings indicate the possibility of gene mutations by a DNA lesion toxin synthesized by *Campylobacter*.

## 4 Discussion

In this study, we report the relationship between host cancer cell genetic abnormalities and the composition of the microbiome present in the tumor tissue in 29 cases of CRC, based on paired data analysis of the cancer genome and microbiome. We made three main observations in this analysis. First observation was that 12 genera, including *Fusobacterium, Peptostreptococcus*, and *Campylobacter*, were found to be positively enriched in the tumor tissue, while 11 genera, including *Bacteroides* and *Clostridium*, were found to be depleted in the tumor tissue. *Fusobacterium* and *Bacteroides* have already been reported to be positive markers and enriched in CRC gut microbiome [42]. Our results also showed that the relative abundance of both these genera was high. Interestingly, *Fusobacterium* was enriched in tumor tissue, whereas *Bacteroides* were enriched in non-tumor tissue. This suggests that different mechanisms of invasion into tumor tissue may be involved, and that *Fusobacterium* was more strongly associated with the tumors in this study. In addition, Zeller et al. reported that *Fusobacterium* and *Peptostreptococcus* were biomarkers of early-stage CRC as compared with the healthy controls [43], and our findings suggested that these two bacteria could also be used as biomarkers distinguishing tumor tissue from non-tumor tissue. Second observation was that *Fusobacterium* was associated with alterations in genes such as *ATM* and *PIK3CA*, and was also associated with gene alterations in the cell cycle system. Third observation was that the *Campylobacter*-high group had a much higher percentage of Signature 3 than the *Campylobacter*-low group, suggesting that the *Campylobacter*-high group had undergone genetic alterations by a DNA lesion toxin synthesized by *Campylobacter*.

*Fusobacteria* are gram-negative anaerobic bacilli with species-specific reservoirs in the human mouth, gastrointestinal tract, and elsewhere [44], [45], [46], [47], [48], [49], [50]. Previous reports have identified associations between composition of the gut microbiome and the risk of CRC, and *F*. *nucleatum* has emerged as an important player in carcinogenesis in CRC. To date, numerous studies of multiple cohorts of patients with CRC have found that *F*. *nucleatum* DNA and RNA sequences were more abundant in tumor specimens than in non-tumor specimens [44]. Evidence suggests that *F*. *nucleatum* may contribute to disease progression and is associated with specific molecular features. Mima et al. reported that a high prevalence of *F*. *nucleatum* was associated with poor differentiation, advanced disease stage, and cancer-specific survival [45], suggesting a specific colorectal carcinogenic effect of *F*. *nucleatum*. Ito et al. reported that a high prevalence of *F*. *nucleatum* was associated with *MLH1* methylation, CpG island methylator phenotype (CIMP), and microsatellite instability-high (MSI-H) in CRC [51], suggesting that *F*. *nucleatum* is associated with specific molecular features in CRC.

ATM encoded by this gene belongs to the PI3/PI4-kinase family. ATM is an important cell cycle checkpoint kinase, which regulates a wide variety of downstream proteins, including tumor suppressor proteins p53 and BRCA1, checkpoint kinase CHK2, checkpoint proteins RAD17 and RAD9, and DNA repair protein NBS1. ATM and ATR are closely related, and control cell cycle checkpoint signaling pathways, which are required for cell response to DNA damage and genome stability. In this study, we found that *Fusobacterium* was significantly associated with genetic alterations in *ATM* and *PIK3CA* in CRC. Lee et al. evaluated the relationship between *F*. *nucleatum* and genetic alterations in the tumor tissue of CRC, and reported that the alteration rate of *ATM* was higher in *F*. *nucleatum*-high patients [52], [53]. Moreover, we found that *Fusobacterium* was associated with gene alterations in the CRC cell cycle system in CRC. The genes involved in cell cycle system that were associated with *Fusobacterium* were “*ATM*”, “*ATR*”, “*CCNE1*″, ”*EP300*″, “*TP53*″, ”*RB1*″, “*CDKN1B*”, and “*CDKN2B*”. Taken together, these findings indicate that *F*. *nucleatum* may lead to tumorigenesis by causing genomic instability, which results from abnormalities in the cell cycle system. However, to the best of our knowledge, there are no reports regarding the association between *F*. *nucleatum* and *PIK3CA* alterations in CRC. We believe that further analysis of the relationship between the microbiome and tumorigenesis of CRC is necessary.

Various intestinal microbiomes have been associated with the development of CRC; however, the direct role of bacteria in oncogenic mutations has not been demonstrated so far. Mutational signatures are characteristic combinations of mutation types arising from specific mutagenic processes. >50 mutational signatures have been defined using mutational signature analysis that includes the bases immediately 5′ and 3′ to a single-base substitution [19]. Although the underlying causes of some mutational signatures are known (e.g., tobacco smoke, UV light, and DNA repair deficiency), those of many mutational signatures are still unknown. To date, there is no evidence that specific bacteria are associated with specific mutational signatures, such as smoking and COSMIC mutational signature 4. However, interestingly, Pleguezuelos-Manzano et al. demonstrated that the distinct mutational signature in CRC was caused by genotoxic *pks + E. coli*, implying that the underlying mutational process results directly from past exposure to bacteria carrying the colibactin-producing *pks* pathogenicity islands [54].

*Campylobacter* is widespread in developed countries, and human infections can result in an asymptomatic carrier state [55], [56]. Interestingly, co-occurrence of *Fusobacterium* and *Campylobacter* has been observed in patients with CRC, as has been an increased prevalence of *Escherichia* and *Campylobacter* in tumor tissue compared with adjacent non-tumor tissue [18]. He et al. reported that *C. jejuni* promotes CRC through the genotoxic action of *cdtB*, which has DNAse activity and causes DNA double-strand breaks. Moreover, pharmacological inhibition of mammalian targets of rapamycin signaling attenuates *C. jejuni*-induced carcinogenesis [18]. In this study, we found that *Campylobacter* was associated with genetic alterations such as *TSC2*, *AR*, *HRAS*, *FGFR3*, and *AKT1*, and patients with a high abundance of *Campylobacter* showed Signature 3. Signature 3 is associated with the failure of DNA double-strand breaks by homologous recombination. We demonstrated that the *Campylobacter*-high group was associated with a much higher percentage of Signature 3, suggesting that *Campylobacter* may promote CRC by causing DNA double-strand breaks.

This study has several limitations. Firstly, it included a small number of patients. Secondly, we performed NGS to detect genetic alterations in tumor tissue using FFPE samples, while 16S rRNA gene sequencing was performed using fresh frozen samples. The difference in sampling sites between NGS and 16S rRNA can affect the results because of tumor heterogeneity. Thirdly, although we demonstrated the association between intra-tumor microbiomes and genetic alterations in this analysis, it is unclear whether the changes in the gut microbiome cause or result from sporadic CRC. In the future, we need to uncover the specific mechanisms involved in the modulation of the gut microbiome by integrated large-scale prospective studies.

## 5 Conclusions

Some bacteria, including *Fusobacterium*, which is already known to be enriched in CRC, were found to be enriched in symbiosis with tumor tissue compared to non-tumor tissue. Furthermore, it was shown that the microbiota often coexisting within tumor tissue was enriched in the presence of an altered gene or an abnormal signaling pathway in the host cells. These results indicate that CRC development may be partially caused by DNA damage or abnormal signaling pathways, caused by substances released by bacteria in the microbiota. Taken together, the identification of distinct gut microbiome patterns and their specific genetic alterations might facilitate targeted interventions, such as modulation of the microbiome in addition to anticancer agents and/or immunotherapy.

## CRediT authorship contribution statement

**Shujiro Okuda:** Investigation, Methodology, Software, Funding acquisition, Writing - original draft, Project administration. **Yoshifumi Shimada:** Methodology, Data curation, Writing - original draft. **Yosuke Tajima:** Methodology, Data curation. **Kizuki Yuza:** Data curation. **Yuki Hirose:** Data curation. **Hiroshi Ichikawa:** Data curation. **Masayuki Nagahashi:** Data curation. **Jun Sakata:** Data curation. **Yiwei Ling:** Software, Visualization. **Nobuaki Miura:** . **Mika Sugai:** Data curation. **Yu Watanabe:** Software, Visualization. **Shiho Takeuchi:** Data curation. **Toshifumi Wakai:** Data curation, Conceptualization, Funding acquisition, Project administration.

## Declaration of Competing Interest

The authors declare that they have no known competing financial interests or personal relationships that could have appeared to influence the work reported in this paper.
